# The Treatment of Iritis

**Published:** 1907-06-15

**Authors:** 


### THE TREATMENT OF IRITIS.

There are so many pitfalls in the treatment of diseases of the eye that some general practitioners have for years disclaimed all knowledge of ophthalmic work and have been content to place these cases under the care of others who make the study of ophthalmic surgery a speciality.

With regard to iritis, however, once the diagnosis is correctly made, the treatment is continued on more or less stereotyped lines, in some cases with rapid success, whilst in others the impossibility of treating the primary cause precludes all probability of a satisfactory result even in skilled hands.

In diagnosis the difficulty lies in discriminating between cases of iritis and those of conjunctivitis and inflammatory glaucoma. In an inflamed eye the condition of the pupil should be very carefully investigated; if the pupillary area is perfectly black when viewed by ordinary daylight, and the pupil reacts evenly and at once to alterations in the intensity of light, the case is unlikely to be iritic.

With regard to glaucoma, it is not sufficiently recognised how rarely patients under 40 years of age are attacked by glaucoma, and how infrequently acute iritis attacks patients over 40 for the first time. Iritis is mainly a disease of young male adults; glaucoma is mainly a disease of middle-aged women.

Atropine should be used in cases of iritis, until the pupil is fully dilated or until the drug ceases to produce any further dilatation. A solution of sulphate of atropine (gr. iv. ad ℥j.) usually produces the maximum effect if instilled liberally at short intervals. The use of the pure sulphate of atropine in the form of crystals placed inside the conjunctival sac does not produce any better effect than that obtained by the gradual dilatation effected by the 4 gr. ad 1 oz. solution applied over a longer period, and the latter has the advantage that it is less likely to produce toxic symptoms.

When using the solid crystals the lachrymal duct must be kept closed by pressure. The use of atropine must be continued as long as the eye shows any inflammatory symptoms, and for absolute safety a few days longer. It is not, however, necessary to use a stronger solution than is just sufficient to keep the pupil dilated after full dilatation has been obtained. A solution of 2 gr. to the ounce is usually amply sufficient for this, if instilled twice or thrice daily.

If atropine irritation ensues the drug should be discontinued and homatropine and cocaine substituted. Duboisine sulphate and scopolamine hydrobromate in 1 per cent, solutions are also mydriatics which can be used instead of atropine.

The reason for using a mydriatic in iritis is that by it the hyperæmia is diminished and the adhesions or synechiæ between the iris and the anterior surface of the lens are broken down.

If the iritis can be traced to a definite and specific poison the treatment will naturally be materially assisted by administering appropriate remedies for the cause: salicylates and aspirin for rheumatic cases, mercury for syphilis, chaulmoogra oil for leprosy, etc.; but there are many cases where a definite specific cause cannot be ascertained, so that the so-called idiopathic cases still remain the larger class. In these cases mercury is always indicated, since it is an antiseptic, and iritis always suggests the presence of a poison within the system; mercury has also an undoubted influence on all inflammatory conditions, whatever their cause.

Diaphoresis combined with mercurial inunction is most valuable in all syphilitic and idiopathic cases. The patient should be recommended to take three very hot baths each week just before bedtime, and to continue these for a period of three or four weeks; after the bath a drachm of the ung. hydrarg. oleatis should be rubbed into the skin until the ointment disappears. The patient should be warned against rubbing the ointment into the same place more than once a fortnight. The armpits, bends of elbows and knees, the groins, and small of back are all suitable places for applying the drug.

In the early stages of acute iritis, especially of the rheumatic variety, pain is often severe. A solution of dionine 1 to 2 per cent., combined with cocaine and adrenalin instilled into the conjunctival sac, is very useful for this symptom. Leeches, or small blisters, applied to the temple sometimes act like magic, and hot lotions are also beneficial.

Cases of iritis, especially those occurring in men accustomed to an active independent life, often produce a very irritable condition of mind, followed by great depression, the result of the pain, partial blindness, and resultant dependence on others. To combat this the patient should be allowed carriage exercise, and be kept as far as possible in cheerful surroundings. The photophobia is sometimes very distressing, but in no case should all light be cut off from the sick room. Smoked glasses and a broad shade should be sufficient to protect the patient, and for the sake of the nurse and the patient's friends, as well as for his general health, the room should not be in total darkness.

All stimulants should, as far as possible, be avoided, as well as meat. The diet should be liberal, but easily digestible. Constipation must be avoided, and treatment should start with a brisk purge.

